# Magnetocontrollability of Fe_7_C_3_@C superparamagnetic nanoparticles in living cells

**DOI:** 10.1186/s12951-016-0219-4

**Published:** 2016-08-30

**Authors:** Irina B. Alieva, Igor Kireev, Anastasia S. Garanina, Natalia Alyabyeva, Antoine Ruyter, Olga S. Strelkova, Oxana A. Zhironkina, Varvara D. Cherepaninets, Alexander G. Majouga, Valery A. Davydov, Valery N. Khabashesku, Viatcheslav Agafonov, Rustem E. Uzbekov

**Affiliations:** 1 A.N. Belozersky Institute of Physico-Chemical Biology, Moscow State University, Moscow, Russia 119992; 2 Biology Faculty, Moscow State University, Moscow, Russia 119992; 3 GREMAN, UMR CNRS 7347, Université François Rabelais, 37200 Tours, France; 4 Chemistry Faculty, Moscow State University, Moscow, Russia 119992; 5 MISiS, Leninskiy prospekt 2, Moscow, Russia 119049; 6 Institute of High Pressure Physics RAS, Troitsk, Moscow region Russia 142190; 7 Center for Technology Innovation, Baker Hughes Inc., Houston, TX 77040 USA; 8 Laboratoire Biologie Cellulaire et Microscopie Electronique, Faculté de Médecine, Université François Rabelais, 37032 Tours, France; 9 Faculty of Bioengineering and Bioinformatics, Moscow State University, Moscow, Russia 119992

**Keywords:** Superparamagnetic nanoparticles, Living cells, Magnetocontrollability, Endocytosis, Cytoskeleton, Cell adhesion

## Abstract

**Background:**

A new type of superparamagnetic nanoparticles with chemical formula Fe7C3@C (MNPs) showed higher value of magnetization compared to traditionally used iron oxide-based nanoparticles as was shown in our previous studies. The in vitro biocompatibility tests demonstrated that the MNPs display high efficiency of cellular uptake and do not affect cyto-physiological parameters of cultured cells. These MNPs display effective magnetocontrollability in homogeneous liquids but their behavior in cytoplasm of living cells under the effect of magnetic field was not carefully analyzed yet.

**Results:**

In this work we investigated the magnetocontrollability of MNPs interacting with living cells in permanent magnetic field. It has been shown that cells were capable of capturing MNPs by upper part of the cell membrane, and from the surface of the cultivation substrate during motion process. Immunofluorescence studies using intracellular endosomal membrane marker showed that MNP agglomerates can be either located in endosomes or lying free in the cytoplasm. When attached cells were exposed to a magnetic field up to 0.15 T, the MNPs acquired magnetic moment and the displacement of incorporated MNP agglomerates in the direction of the magnet was observed. Weakly attached or non-attached cells, such as cells in mitosis or after cytoskeleton damaging treatments moved towards the magnet. During long time cultivation of cells with MNPs in a magnetic field gradual clearing of cells from MNPs was observed. It was the result of removing MNPs from the surface of the cell agglomerates discarded in the process of exocytosis.

**Conclusions:**

Our data allow us to conclude for the first time that the magnetic properties of the MNPs are sufficient for successful manipulation with MNP agglomerates both at the intracellular level, and within the whole cell. The structure of the outer shells of the MNPs allows firmly associate different types of biological molecules with them. This creates prospects for the use of such complexes for targeted delivery and selective removal of selected biological molecules from living cells.

**Electronic supplementary material:**

The online version of this article (doi:10.1186/s12951-016-0219-4) contains supplementary material, which is available to authorized users.

## Background

The studies of interaction mechanisms between various types of MNPs and living cells, as well as internalization routes and intracellular motility of individual magnetic particles or their agglomerates are very important for development of various biotechnological applications of magnetic nanomaterials [1–4]. One of the applications of MNPs in cell biology is magnetofection—magnetic field-driven delivery of cargo-loaded MNPs through the cellular membrane. The magnetofection is already a widespread approach to an accelerated transport of nucleic acids associated with MNPs into cells by a magnetic field [3, 5, 6]. However, the precise details of MNPs behavior in living cells and the possibility of their subsequent removal from the cells under the effect of magnetic field still remains an open question.

Generally, the behavior of MNPs in homogeneous liquids under the effect of external magnetic fields is currently well characterized [7, 8]. Since superparamagnetic MNPs are mono-domain magnets, the magnetic dipole–dipole interaction between them in the constant homogeneous magnetic field should induce their alignment along the magnetic field lines of the permanent magnet. The magnetic attraction force in anisotropic magnetic field causes MNPs acceleration in the direction of increasing magnetic field strength. This force, which is typically a few pN for MNPs [8], is proportional to the magnetic field gradient, MNP volume and its magnetic moment. The alignment and movement speed of MNPs also depend on the viscosity of the medium and hydrodynamic radius of the nanoparticle [7, 8].

In contrast to liquid, the behavior of MNPs in living cells is also affected by anisotropic viscosity and resilience of the cytoplasmic structures, such as cytoskeleton and different membranous compartments. The main process of MNPs internalization into the cells is known to be an endocytosis [9, 10]. The intracellular MNPs may exist in various states like free individual particles or their agglomerates of 100–200 nm in diameter, or enclosed in membrane vesicles—so-called «magnetic endosomes» [11]. Free cytoplasmic MNPs may originate from the «magnetic endosomes» through the process termed « endosomal escape » [12, 13]. Besides the anisotropic viscosity of cytoplasm and the resilience of the cytoskeleton filaments, the movements of magnetic endosomes can be affected by the activity of cytoskeleton-associated protein motors acting in two opposite directions—kinesin-like motors translocating vesicles from the center of the cell to the periphery (centrifugally) and dynein-like ones acting towards the center (centripetally), as well as actin-associated myosins [14].

It was shown that the relatively low value of magnetization of traditionally used SPIONs creates difficulties for the control of their magnetic behavior in a number of applications. A new type of superparamagnetic nanoparticles with chemical formula Fe_7_C_3_@C was recently obtained by high pressure and high temperature process and studied by physico-chemical and biological methods [15, 16] (Table 1). The in vitro biocompatibility tests demonstrated that Fe_7_C_3_@C MNPs display high efficiency of cellular uptake and do not affect cyto-physiological parameters of cultured pig kidney epithelia (PK) cells [16].

In present work we performed a study of Fe_7_C_3_@C MNPs behavior in living cells cultured in vitro in the presence or absence of a constant magnetic field to evaluate the impact of cytoskeleton architecture and cell-substrate interactions on their magneto-controllability at cellular and subcellular levels.

**Table 1:** Magnetic properties of various types of MNPs

NP	Size (nm)	Ms (emu/g)	Source
(Fe_7_C_3_@C)	25	54	Our data [16]
Fe_3_O_4_ (+PEG or DOX)	7–10	1.12	[25]
γ-Fe_2_O_3_ @C	15	28	[26]
γ-Fe_2_O_3_ @Si	50–200	15–35	[27]
γ-Fe_2_O_3_ (pure)	15	35	[28]

## Methods

### Cell culture and experimental treatments

Human fibrosarcoma cells HT1080 (kindly provided by Russian Collection of cell lines, St. Petersburg) were cultured in DMEM culture media (Sigma, USA) supplemented with 10 % fetal calf serum (HyClone, USA) and antibiotic–antimycotic (100 units/ml penicillin G, 100 mg/ml streptomycin sulfate and 0.25 mg/ml amphotericin B) (Sigma, USA). For microscopic experiments cells were plated onto cover slips at a concentration of 10,000 cells/cm^2^ and grown for 48 h to reach 50 % confluency before the addition of Fe_7_C_3_@C MNPs to a final concentration of 20 µg/ml. Kinetics of cell interaction with Fe_7_C_3_@C was studied by TEM on serial ultrathin sections, and also by optical microscopy using time lapse video recording of living cells.

### Live cell experiments

For live imaging, human fibrosarcoma cells were plated on glass-bottomed Petri dishes (LabTek, USA) at a density of 10^5^ cells/ml and incubated with Fe_7_C_3_@C MNPs for 24 h. Imaging was performed in an environmental chamber kept at 37 °C under 5 % CO_2_. The chamber was mounted on a Ti-E inverted microscope (Nikon, Japan) equipped with EMCCD-camera iXon (Andor) operating under control of NIS-Elements 4.0 software. Illumination conditions (ND filters, lamp voltage, and exposure time) were set to minimize photo toxicity. To generate a magnetic field we used in our experiments a gold-plated NdFeB permanent magnet with the size of 5 × 5 × 5 mm and B_z_ = 0.15 T. The magnet was placed directly inside the dish, therefore cells located up to 2 mm from the magnet edge have been recorded. Images were taken every 10 min for 72 h for long recording or every 1 min during short recording. Image sequences were analyzed and time-lapse movies of cells loaded with MNPs were assembled using ImageJ software.

### Immunofluorescent staining

For immunofluorescent staining, cells were fixed with 4 % formaldehyde (Sigma) in physiological phosphate buffered saline (PBS), pH 6.8, for 10 min and then rinsed with three changes of PBS (for 10 min each). The fixed cells were permeabilized with 0.1 % Triton X_100 (Sigma) in PBS for 15 min with subsequent washing out with PBS (three times for 10 min). For elimination of background fluorescence, prior to labeling with antibodies, the cells were treated with 0.2 % NaBH_4_ (Sigma) in PBS (three times for 10 min) and rinsed with PBS (three times for 10 min). The cells were then incubated with primary anti-Rab5 (C8B1) rabbit monoclonal antibodies (Cell Signaling, US, dilution 1:100) (30 min, 37 °C) and secondary antibodies conjugated with Texas Red fluorescent dye (Molecular Probes, dilution 1:1000) (30 min, 37 °C). Cells were mounted in Mowiol and observed in Eclipse Ti-E fluorescent microscope (Nikon, Japan) with CFI Plan Apo VC 60X/NA 1.4 lens, equipped with EMCCD-camera iXon (Andor) under the control of NIS-Elements 4.0 software.

### Transmission electron microscopy

For transmission electron microscopy (TEM) experiments, cells were washed three times with fresh pre-warmed media to remove free particles, fixed in 2.5 % glutaraldehyde in 0.1 M phosphate buffer (pH 7.4) for 2 h with subsequent post-fixation in 1 % OsO_4_ and embedding in Epon (Sigma, USA). Serial ultrathin sections (70 nm) were cut with Leica Ultracut-E ultramicrotome and observed with JEM 1011 (JEOL, Japan) equipped with a Gatan digital camera driven by Digital Micrograph software (Gatan, Pleasanton, USA) at 100 kV.

### Correlative Magnetic force microscopy and Transmission electron microscopy (CMFM-TEM)

For CMFM-TEM we used a Solver microscope (NT-MDT) under air conditions to investigate magnetic properties of Fe_7_C_3_@C MNPs. Semi-thin (500 nm) Epon sections of MNPs-loaded cells fixed after 24 h incubation in magnetic field were attached to 5 × 5 mm glass slide (1 mm thick) and measured by the two-pass MFM method using a silicon cantilever with Co-Cr coating (resonant frequency is 62.7 kHz, spring constant (*k*) is 3 N/m, created magnetic moment is about 10^−13^ emu) [17]. During first pass, the morphology of the cell is determined. Then, the cantilever was lifted from the surface and kept at a constant distance of Δz = 100 nm, then by following the surface morphology profile the response of vertical component of magnetic gradient was recorded (i.e. phase shift (Δ*φ*) of the cantilever oscillation). During second pass, the cantilever oscillated with 50 %, reduced amplitude the vibration system quality factor (*Q*) was 10, Δz-magnetic field was around 200 Oe. All 512 × 512 pixels (40 nm/pixel) images were recorded with a scan rate of 0.5 Hz.

After MFM imaging semi-thin sections were reembedded in Epon, ultrathin sections (70 nm) of the same cells were made and imaged with JEM 1011 (JEOL, Japan) equipped with a Gatan digital camera driven by Digital Micrograph software (Gatan, Pleasanton, USA) at 100 kV. Image alignment and scaling was performed with Photoshop CS3 (Adobe, USA).

## Results

### Endocytosis of MNPs by human fibrosarcoma cell

**Fig. 1:**
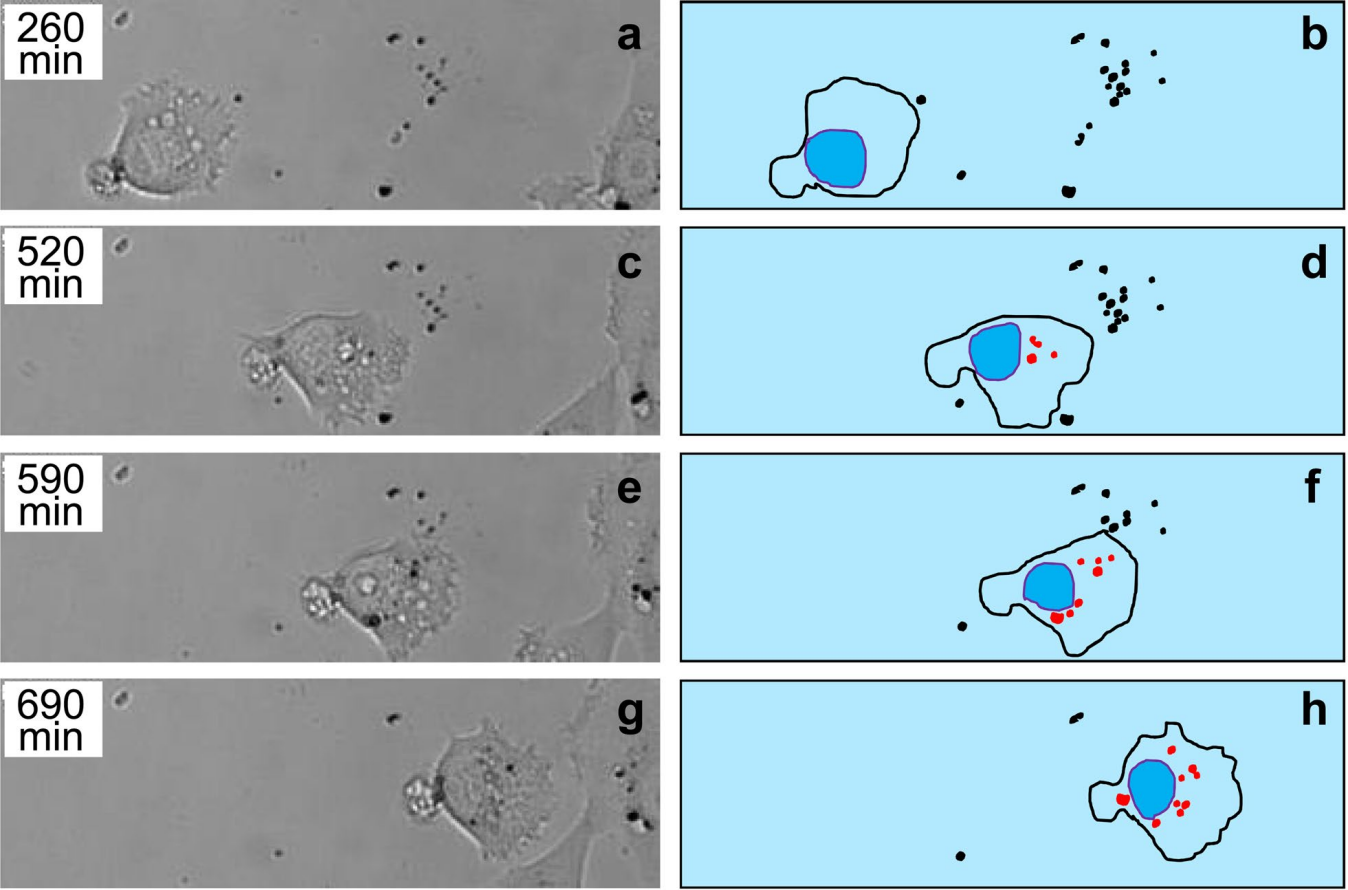
Live imaging of moving HT-1080 cell that actively uptakes MNPs from the glass surface. Time scale from the beginning of the recording is indicated in the *upper-left corner* of each image (*left column*). *Left column* (**a**, **c**, **e**, **g**) represents successive photos of the cell, *right column* (**b**, **d**, **f**, **h**) represents a sketch of the movie with free MNPs shown in *black* and internalized MNPs in *red* (see also Additional file 1: Movie 1)

In the present work we performed live-cell imaging to study the kinetics of interactions between MNPs and transformed cells isolated from biopsies of human fibrosarcoma (line HT1080). The cells grown on solid substrate acquire fibroblast-like shape and demonstrate rather high motility; cell movement speed was measured as about 0.2 µm/min (Fig. 1; Additional file 1: Movie 1). This cell line also demonstrates high proliferative activity typical for transformed cells. During cell division, the cells round up and lose contacts with the substrate, performing all the phases of mitosis (including ana- and telophase) in this rounded state.

After administration of MNPs suspension to the culture media, the cells actively internalize the agglomerates of MNPs formed in solution and on the cell surface by endocytosis, similar to what we described earlier for non-transformed cells [16]. Internalized MNPs move from the cell membrane into the cytoplasm and form one or several agglomerates of various sizes.

Live-cell imaging demonstrated that the cells can actively collect MNPs agglomerates laying on the substrate (Fig. 1; Additional file 1: Movie 1) as well as on the surface of neighboring cells (Additional file 2: Movie 2) during their movement.

The mitotic activity of transformed MNPs-treated fibrosarcoma HT1080 cell line remained the same as in control untreated cells. Abnormal mitotic figures, colchicine-like mitotic cells and cells with chromosome segregation anomalies as well as with cytokinesis defects, were not observed in these experiments. All observations described here allowed us to conclude that MNPs have no cytotoxicity effect on cultured HT1080 cells, similarly to our experiments with MNPs-loaded non-transformed PK cells [16].

### Immunofluorescence analysis of MNPs and endosome co-localization in the cells

**Fig. 2:**
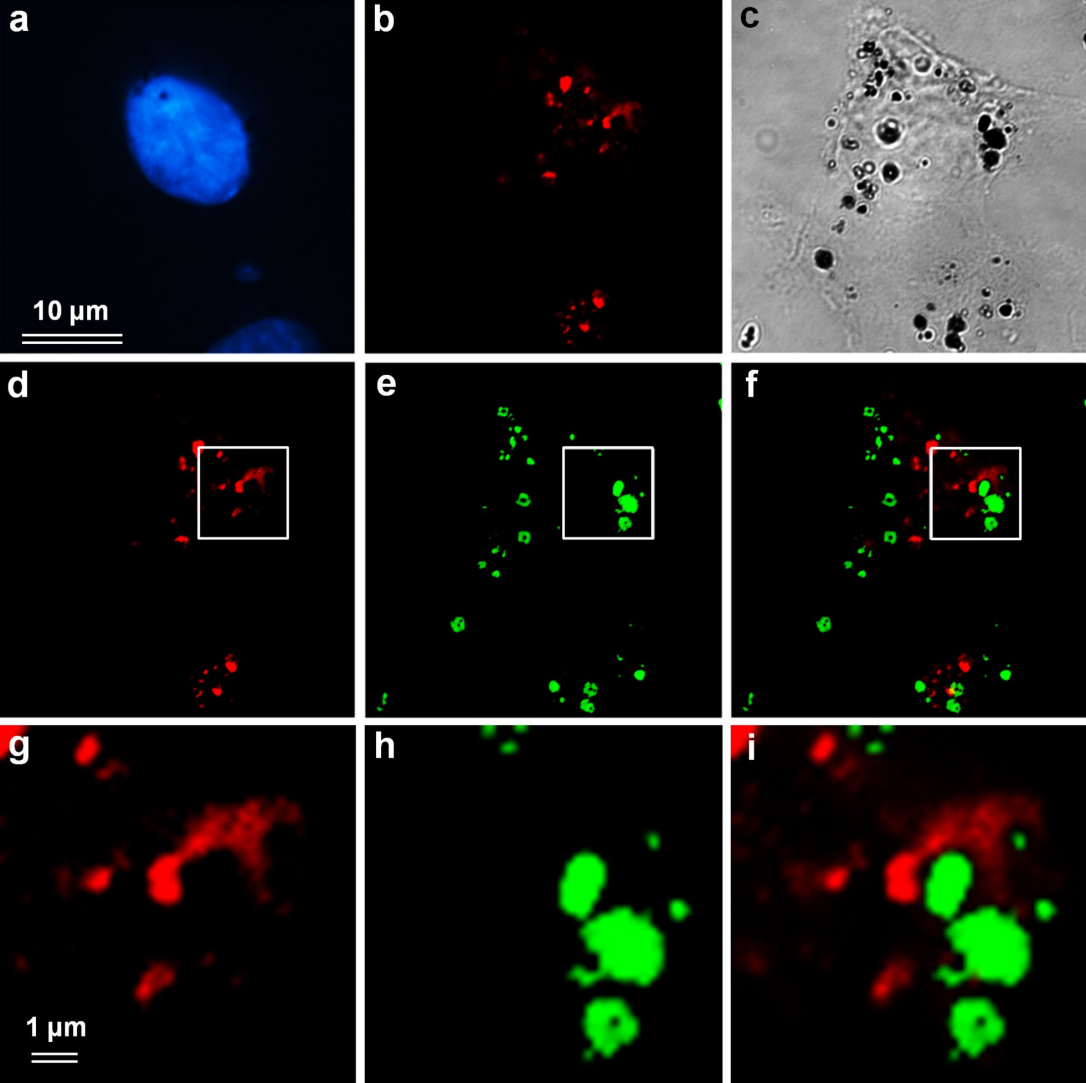
Immunofluorescence analysis of MNPs and endosomes co-localization in the cells. **a** DAPI nuclear labeling, **b**, **d**, **g** endosome visualization with antibodies against Rab5 (*red*); **c** phase contrast, **e**, **h** pseudo color presentation of MNPs localization (*green*), **f**, **i** overlay of endosomes and MNPs. **g**–**i** Show enlarged areas indicated on **d**–**f** with a frame. *Bar* 10 µm (**a**–**f**), 1 µm (**g**–**i**)

In our previous work we suggested that at least part of MNPs is localized inside the endosomes [16, 18]. To confirm these observations we studied colocalization of cytoplasmic agglomerates of MNPs with endosomes immunostained for endosomal marker Rab5 (Fig. 2). Immunofluorescence analysis showed us that the regions of cytoplasm where endosomes are preferentially localized match rather well the area of MNPs agglomerates distribution with some small agglomerates of MNPs located inside the endosomes. However, the majority of endosomes are free of detectable MNP agglomerates and many of the latter, especially big ones, did not colocalize with endosomes either. This observation may suggest that the “endosome escape” occurs rather early, after MNPs internalization, before formation of secondary lysosomes. Otherwise, one would observe high cell mortality due to the membrane destruction and cytoplasmic release of activated lysosomal enzymes.

### Effects of magnetic field on intracellular MNPs positioning and movements

The main motivation of using superparamagnetic nanoparticles in current study was the possibility to manipulate their localization and movement by external magnetic field. Relatively small size of the magnet used allowed its positioning inside a glass-bottomed Petri dish utilized for live imaging, so the cells can be placed in close vicinity to the magnet where the intensity of magnetic field is sufficiently high. Direct measurement of the magnetic fields showed typical exponential attenuation from 0.15 T near the surface to 0.01 T at the distance of 25 mm. All experimental cells we observed were located within 1 mm from the magnet surface, thus the magnetic field intensity at this distance ranged from 0.15 to 0.1 T.

**Fig. 3:**
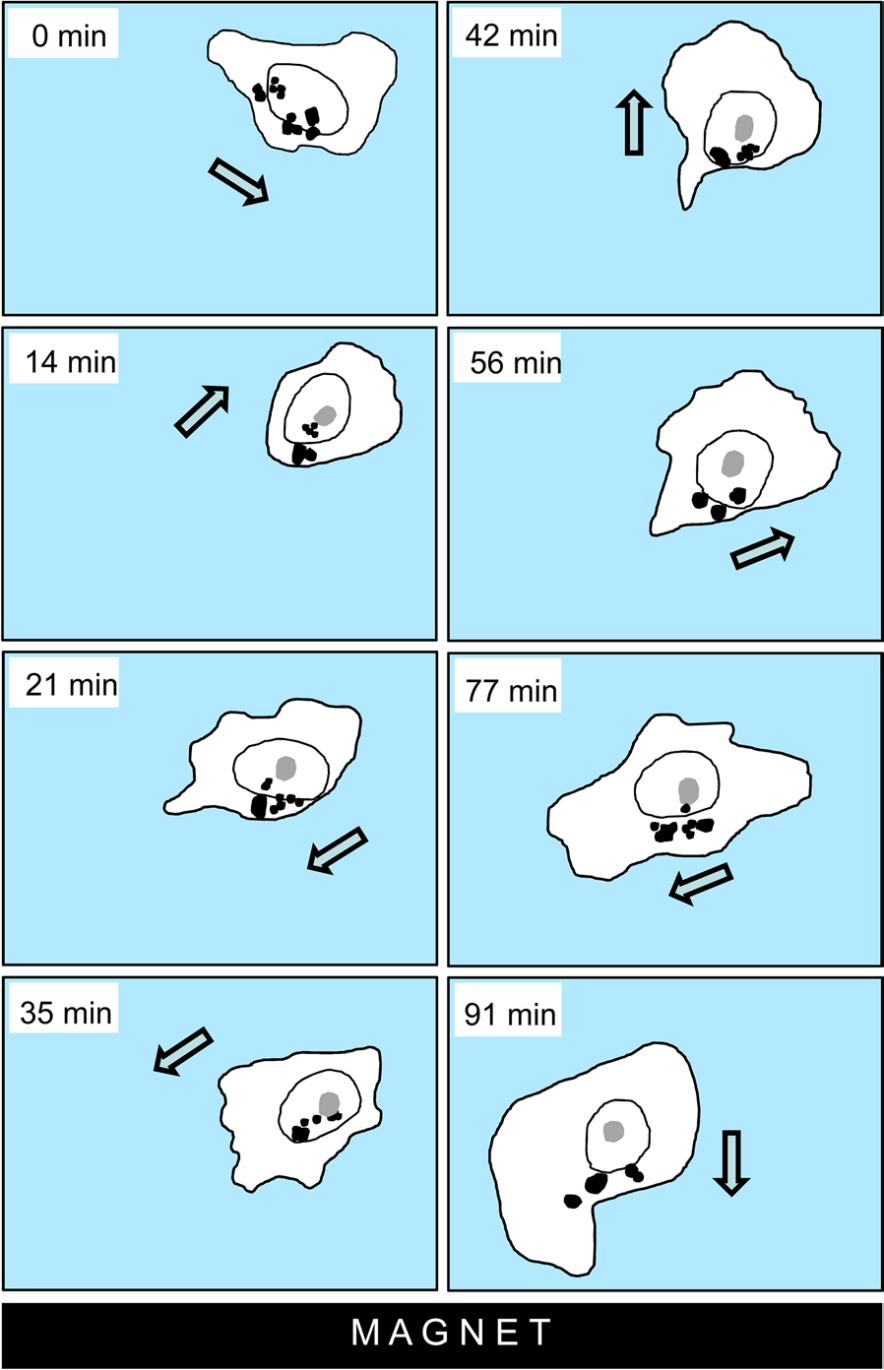
As a cell changes the direction of its movement (the movement direction indicated by an *arrow* on this drawing for each time point), MNPs (indicated by *black dots*) in the cell can keep their position on the side facing the magnet (its position is down in this case). See also Additional file 3: Movie 3

As has been already demonstrated earlier [16], internalized MNPs move from the cell surface into the cytoplasm where they form one or several agglomerates or stay as individual particles. Upon applying an external magnetic field these agglomerates are capable of moving in the direction of the source of magnetic field, i.e. permanent magnet, along the magnetic field lines (Fig. 3; Additional file 3: Movie 3).

This movement is rather slow relative to cell motility so it was impossible to measure momentary speeds. However, after prolonged observations of cells located close to the magnet surface, gradual accumulation of MNPs in the part of the cells facing the magnet has become obvious (Fig. 3). The degree of MNP agglomerate alignment critically depends on cell movement activity: the more actively the cell changes its position and shape, the less MNPs alignment towards the magnet occurs.

Along with a slow drift of internalized MNPs towards the magnet, numerous agglomerates of MNPs tend to orient themselves in magnetic field so they form highly extended agglomerates of smaller MNPs agglomerates with their long axis becoming parallel to the magnetic field lines. This orientation does not typically affect cell motility but is usually preserved upon changes in cellular shape, cytoskeleton rearrangement and changes of the direction of cell migration (Additional file 4: Movie 4).

Live imaging has also showed that, while a control cells move chaotically with respect to the orientation of external magnetic field, the MNPs-loaded cells display different behavior. The cells with high concentration of cytoplasmic MNPs tend to move in the direction of the magnet (Additional file 5: Movie 5). Apparently, the magnetic force in the vicinity of the magnet is high enough to counteract the forces generated by cytoskeleton in moving cells and deviate their trajectory towards the magnet.

### Exocytosis of MNPs out of cell cytoplasm under the action of magnetic field

**Fig. 4:**
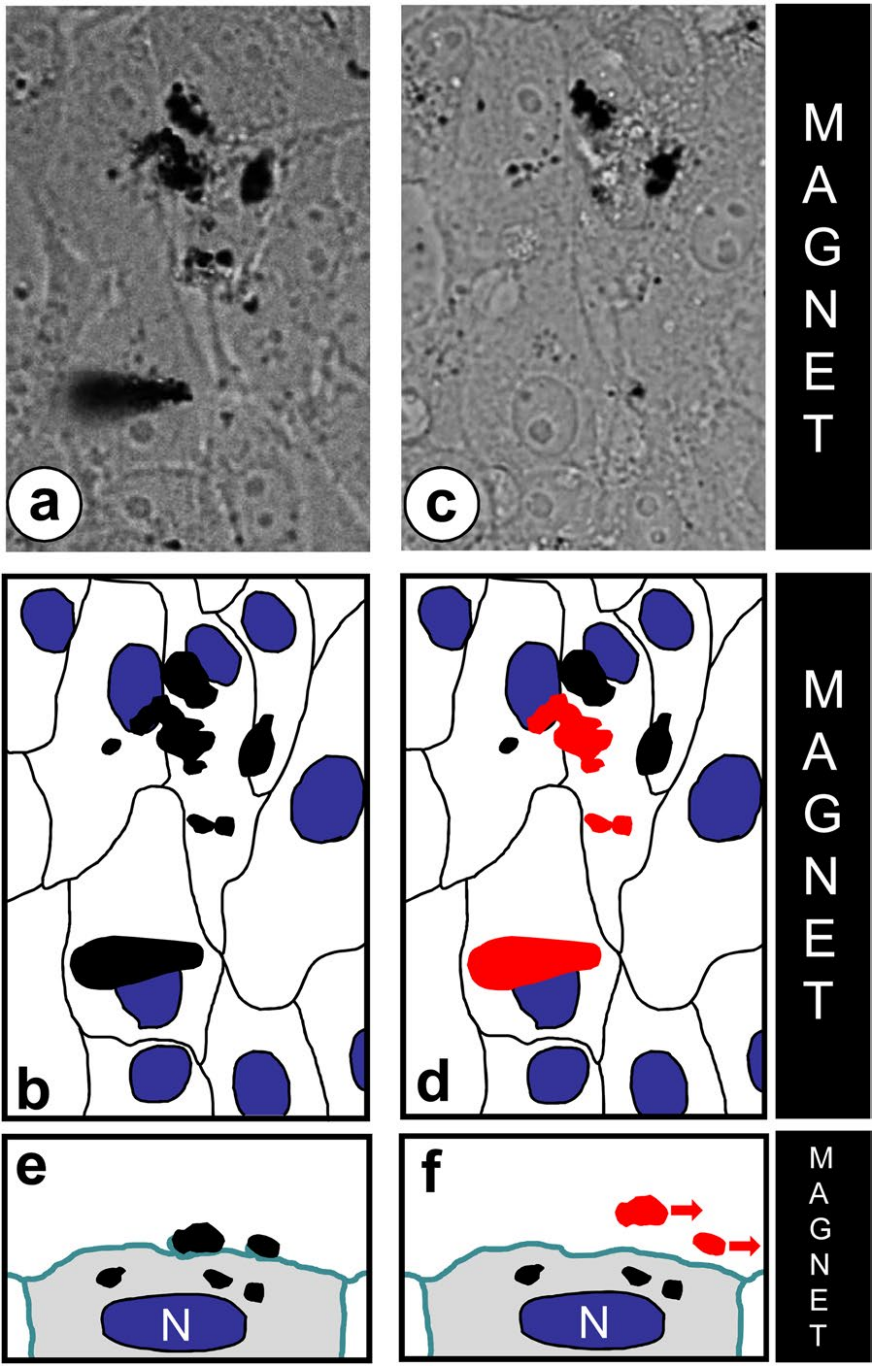
The immediate effect of magnet introduction on MNPs agglomerates on the surface of the cells. **a**–**c** cells after 24 h of incubation with MNPs, **d**–**f** cells 5 min after magnet introduction, **a**, **d** phase contrast images, **b**, **e** schematic drawing of the cells shown in *figure* “**a**, **d**”, **c**, **f** schemes illustrating the effect of magnet on MNPs agglomerates on the cell surface

As shown previously, the MNPs, which adhered to the cell membrane were internalized within 12 h after MNPs addition [16]. Live cell imaging provided several examples of MNPs agglomerates located in cytoplasm, which move towards cell periphery under the effect of magnetic field and eventually pass through a plasma membrane and leave the cell. This process is apparently exerted through exocytosis pathway. Once outside the cell, the agglomerates are rapidly translocated towards the magnet (Fig. 4).

Long-term observations of interactions between HT1080 cells and MNPs have also showed many examples of MNPs «recycling». After losing most of internalized MNPs under the effect of magnetic field the cell can absorb new MNPs, collecting them again either from the substrate or from the surface of neighboring cells (Additional file 2: Movie 2). This «recycling» apparently reflects intermittent or constant endocytotic and exocytotic activities, which involve MNPs available until all of them are removed from culture media by magnetic field.

### Ultrastructure and subcellular distribution of incorporated MNPs

**Fig. 5:**
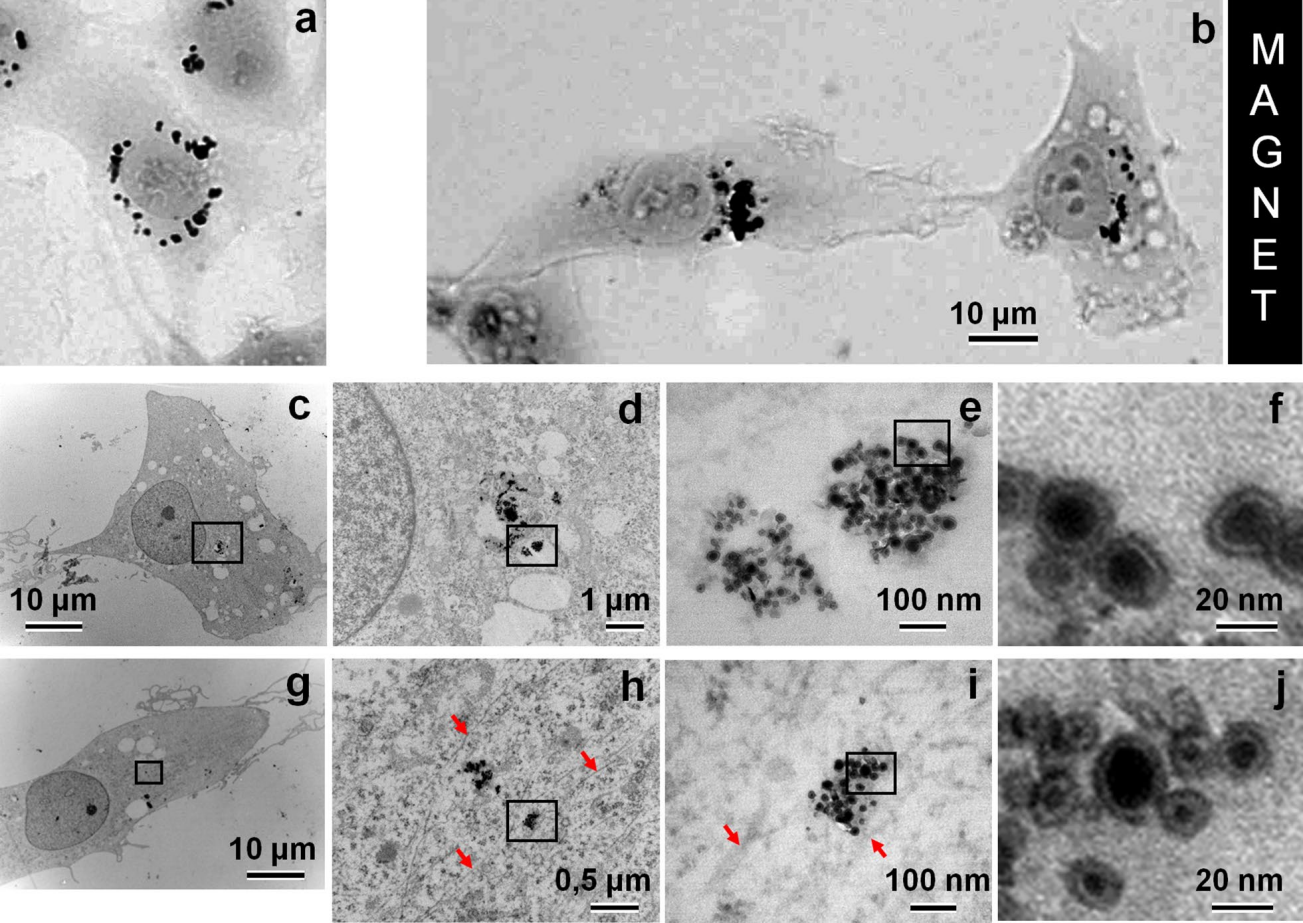
The effect of prolonged exposure (24 h) of magnet on MNPs agglomerates in the cells. **a** Phase contrast images of cells cultivated without magnet, **b** phase contrast images of cells cultivated with magnet placed at *right side* (distance near 2 mm), **c**–**j** correlative electron microscopy pictures of two cells shown on “**b**” at different magnifications. MNPs agglomerates preferably lie freely in the cytoplasm of the cells, a part of agglomerates is in contact with microtubules (*red arrows*). Photos “**f**, **j**” show a primary ultrastructure of MNPs

To better understand the behavior of MNPs inside the cell and estimate the effect of cytoplasmic environment on the structure of MNPs themselves, the cells loaded with MNPs were subjected to TEM analysis. We applied correlative light-electron microscopy approach, selecting those cells which contained large MNPs agglomerates oriented in the direction of magnet. It was found that after internalization, MNPs can be associated with endosomes (Fig. 5d), but more often they break free from endosomes and lay intact in the cytoplasm as single MNPs or groups of various sizes (Fig. 5h, i, arrows). These observations confirmed the immunofluorescent data on incomplete colocalization between MNPs and endosomal marker Rab5 (Fig. 2). MNPs preserve their typical structure of electron-dense metal core surrounded by less dense carbon shell, showing no indications of defects in their shells (Fig. 5f, j). Thus, cytoplasmic environment seems to be permissive to Fe_7_C_3_@C MNPs structure and shell integrity, which explains their extremely low toxicity.

### In situ measurements of magnetic properties of MNPs by CMFM-TEM

**Fig. 6:**
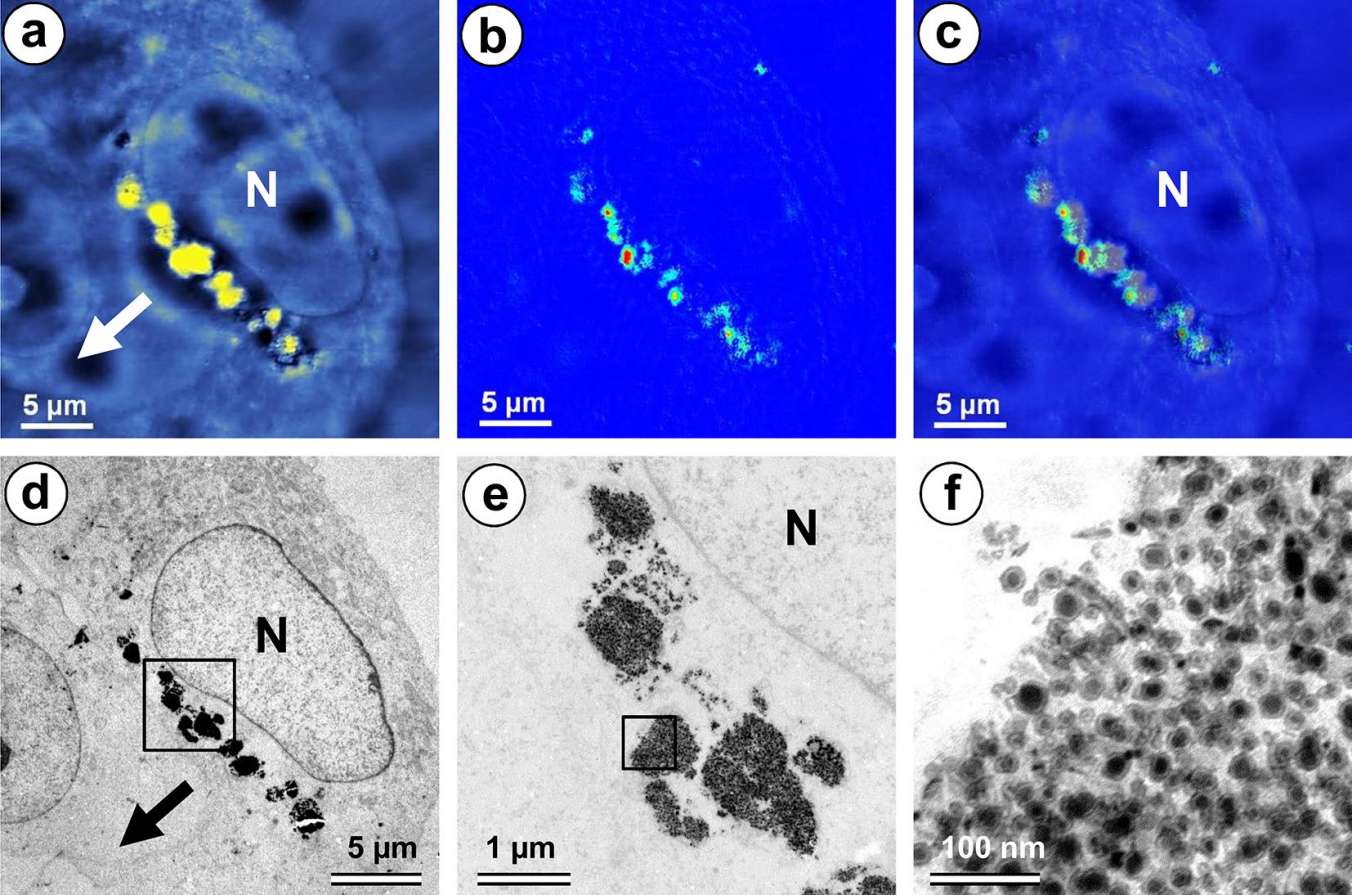
CMFM-TEM microscopy images of the cell with Fe_7_C_3_@C MNPs taken after the 24 h exposure to magnetic field. **a** Morphology image of the cell in AFM; **b** phase shift map; **c** overlay image of superimposed topographic and phase shift map; **d** the same field of view in TEM; **e** group of Fe_7_C_3_@C MNPs agglomerates; **f** fine ultrastructure of agglomerate region with highest magnetic moment. *N* the cell nucleus. An *arrow* indicates the direction towards the magnet. *Scale bar* **a**–**d** 5 µm, **e** 1 µm, **f** 100 nm

In order to be able to move in a magnetic field, the superparamagnetic Fe_7_C_3_@C MNPs should acquire magnetic polarization. To estimate a degree of this process for MNPs located inside the cells, we performed correlative MFM-TEM microscopy. The magnetic phase contrast image shows the gradient of magnetic fields and allows us to conclude that Fe_7_C_3_@C MNPs agglomerates clearly exhibit magnetic properties (Fig. 6a–c). Superimposed images do not show any significant correlation between the morphology and the magnetic response (Fig. 6c) indicating that each agglomerate contributes individually to the resulting magnetic moment. The force gradient was found to be *kΔφ/Q* ≈ 2.01 N/m and the resulting magnetic moment was estimated to be about 8.4·10^−14^ emu (evaluation based on [19, 20]). The preliminary application of an external magnetic field thus led to the orientation and alignment of Fe_7_C_3_@C MNPs agglomerates.

### Are the components of the cell cytoskeleton—microtubules and microfilaments—involved in the response of MNPs to magnetic field influence?

A closer look at MNPs in magnetic field-oriented agglomerates showed their close apposition to microtubules (Fig. 5h, i). We can speculate that, provided our earlier observations of saltatory movement of MNPs [16] and relatively minor effects of magnetic field on their intracellular movement compared to free MNPs in solution (this study), both membrane-encircled and non-endosomal MNPs can interact with microtubules through some cross-linkers or motor proteins. Our speculations led us to a hypothesis that restricted motility of MNPs in the cytoplasm, caused by their binding to cytoskeletal structures rather than sole viscosity of the cytoplasm, can be facilitated upon selective disruption of microtubules or actin filaments. To test this hypothesis, we performed live cell imaging of MNPs-loaded cell placed in magnetic field in the presence of microtubule-depolymerizing drug nocodazole or cytochalasin D, which disrupts actin cytoskeleton. Quite unexpectedly, we did not observe a dramatic increase in MNPs distribution under the effect of magnetic field after disassembly of either type of the cytoskeleton. This suggests that both microtubules and actin filaments are involved in specific or non-specific interaction with MNPs in the cytoplasm. However, simultaneous treatment with both nocodazole and cytochalasin D caused a cells round-up and loss of contact with the substrate. Under these conditions, application of the magnetic field led to rapid and massive displacement of entire cells (either individual cells or cellular agglomerates) towards the magnet (Additional file 6: Movie 6). Speed of this replacement was critically depend on degree of detachments of the cells from support and vary from 2 to 13 µm/min.

**Fig. 7:**
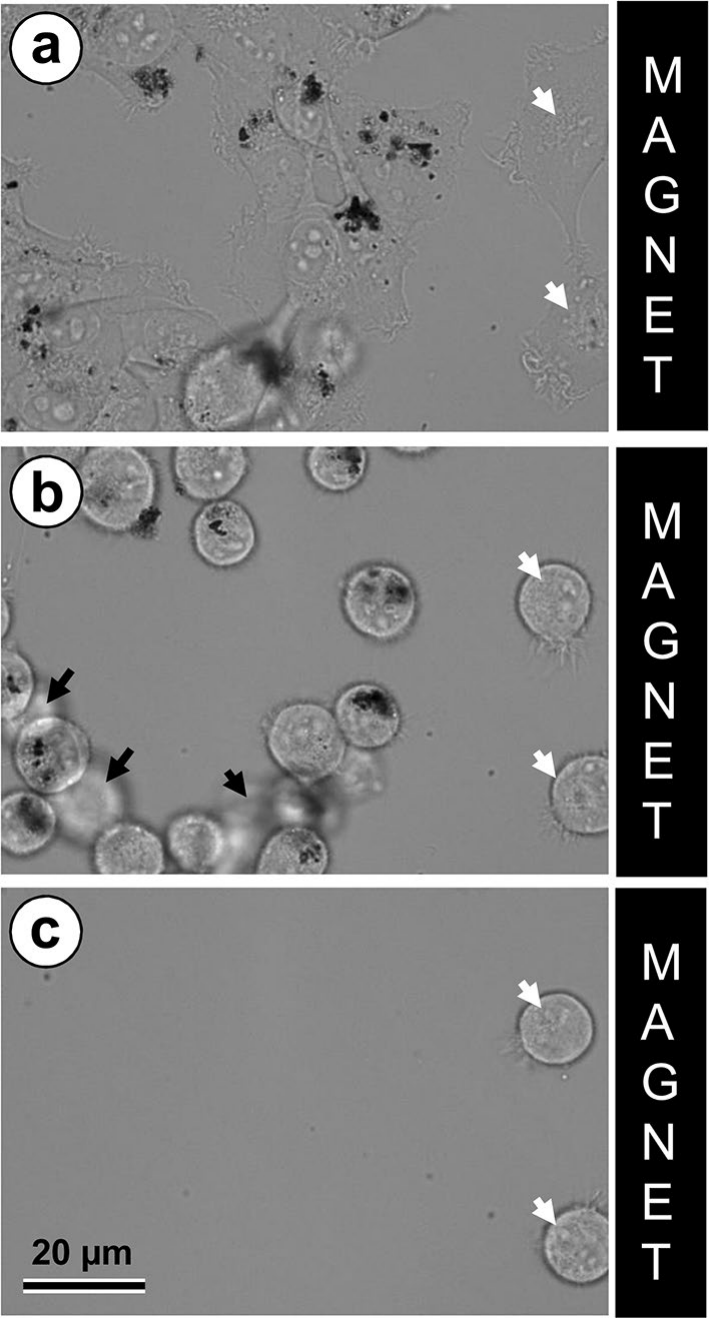
Phase contrast images of cells movement in magnetic field after EDTA treatment. MNP-containing cells detached and moved to the magnet. Cells without MNP (marked with *white arrows*) detached but kept their positions. Cells shown in the fig. **b** (marked with *black arrows*) were moving to the magnet from left part of the sample. **a** Cells before EDTA treatment; **b** 30 min of EDTA treatment; **c** 100 min of EDTA treatment. *Scale bar* 20 µm

Similar effect has been observed after treatment with Ca^2+^ chelator EDTA which stimulates detachment of cells from the substrate (Fig. 7). It must be noted that cells lacking internalized MNPs rounded up the same way as other cells but did not move towards the magnet (Fig. 7c).

## Discussion

The main idea of using MNPs as a tool for intracellular manipulations requires the MNPs to comply certain requirements, including cell permeability, low toxicity and magnetocontrollability. This latter property, apart from achieving principle goals of positioning and/or moving MNPs inside the cell, is particularly useful for removing MNPs at the end of their action, thus further improving their biocompatibility on the organismal level.

Since unmodified carbide MNPs are efficiently internalized by non-transformed cells and are non-toxic for them [16], we anticipated that the same properties are characteristic for their interaction with transformed cells as well. Indeed, the kinetics of MNPs internalization by human fibrosarcoma cells in vitro and cell viability tests gave results practically identical to previously described experiments on PK cells.

As has been reported in our previous work, the main mechanism of MNPs internalization is endocytosis. However, detailed ultrustructural analysis of intracellular localization of MNP agglomerates demonstrated that the majority of MNPs are either only partially encircled by the membrane or lay free in cytoplasm. These results were confirmed by the immunostaining for late endosome marker Rab5. The absence of colocalization apparently suggests that the vesicles containing MNPs are disrupted shortly after internalization. Observations of endosome escape with similar kinetics have been reported for nanodiamonds [21]. Since the nanodiamonds and Fe_7_C_3_@C MNPs possess identical carbon surface structure, this behavior is not surprising. However, in contrast to the hypothesis explaining fast endosome escape of nanodiamonds by the mechanical damage of membranes due to prickly shape of nanodiamonds [21], we believe that fast MNPs release into the cytoplasm depends on the chemical structure of the particles but not their shape. The mechanism of carbon nanoparticles release ([21] and this study) apparently differs from phagocytosis pathway typical for internalization of biological substances (bacteria, cell debris, etc.) [12, 22]. Early MNPs release from immature endosomes does not provoke spillage of lysosome enzymes in the cytoplasm, which explains the absence of detectable cytotoxicity of Fe_7_C_3_@C MNPs.

Measurements of Fe_7_C_3_@C MNPs magnetic properties demonstrated their superparamagnetic capacity [16]. On the other hand, low intrinsic toxicity of carbon surface of Fe_7_C_3_@C MNPs does not require additional protective coating being necessary for SPION nanoparticles [23]. Therefore, the magnetic properties of Fe_7_C_3_@C MNPs are far superior compared to biocompatible SPIONs, thus opening a new window for intracellular magnetocontrollability. Here we tested the effect of constant magnetic field on distribution and movement of the MNPs in living cells. Relatively low intracellular mobility of the MNPs compared to their in vitro behavior in magnetic field can obviously be explained by high viscosity of cytoplasm; however, cytoplasmic environment cannot be approximated as merely homogenous concentrated solution of biopolymers. The behavior of MNPs depends to a great extent on their subcellular localization, overall cytoskeleton organization and a mode of MNPs interaction with main cytoskeletal systems (microtubules, actin and intermediate filaments). Importantly, this slow movement of cytoplasmic MNPs compared to MNP agglomerates in solution, and variations in the mode of MNPs motility inside the cell (Brownian motion and salutatory movements observed in the same cell) suggest involvement of cytoskeleton. Indeed, an association of cytoplasmic MNP agglomerates has been observed in our study at the ultrastructural level at least with microtubule cytoskeleton system. These observations suggest the involvement of microtubule-associated protein motors (dyneins and kinesins) in directional movement of Fe_7_C_3_@C MNPs. However, whether these movements involve vesicle associated MNPs or direct interaction of MNPs with microtubules requires further investigation. We also cannot exclude that other cytoskeletal systems, including intermediate filaments which are more rigid and hard to experimentally disassemble in living cells, may affect passive or active microrheology of nanoparticles in living cells.

Nevertheless, even moderate magnetic fields (less than 0.15T) applied to the internalized agglomerates of Fe_7_C_3_@C MNPs, induce their magnetic polarization, as seen by correlative MFM-TEM, and are capable of alignment of the MNPs along magnetic field lines and concentration at magnet-proximal side of the cell. Similar alignment of natural “magnetosomes”, made by magnetotactic bacteria has been described earlier [24]. Although the preliminary application of an external magnetic field leads to localization of nanoparticles in the cell and does not participate in magnetic properties of individual superparamagnetic nanoparticles imaged by MFM, formation of large agglomerates of Fe_7_C_3_@C MNPs causes residual magnetization. This effect depends on the size of the agglomerate, which explains more visible phase shift for larger agglomerates when analyzed by CMFM-TEM (see Fig. 6).

The specific order of MNPs distribution imposed by external magnetic field can be often counteracted by active cellular motility. This balance of forces was critically dependent of the amount of internalized MNPs. These effects hold true primarily for the cells attached to a substrate, and the situation dramatically reverses when cells loose adhesion, due to either cytoskeleton depolymerization or modification of Ca^2+^-dependent adhesive properties of cellular membrane, as shown in our experiments. In these conditions, the magnetic force was sufficient to fast and immediate translocation of entire cell containing MNP agglomerates towards the magnet. Similar effect was observed for MNP agglomerates positioned on the cell surface immediately upon introduction of magnetic field (Fig. 4).

**Fig. 8:**
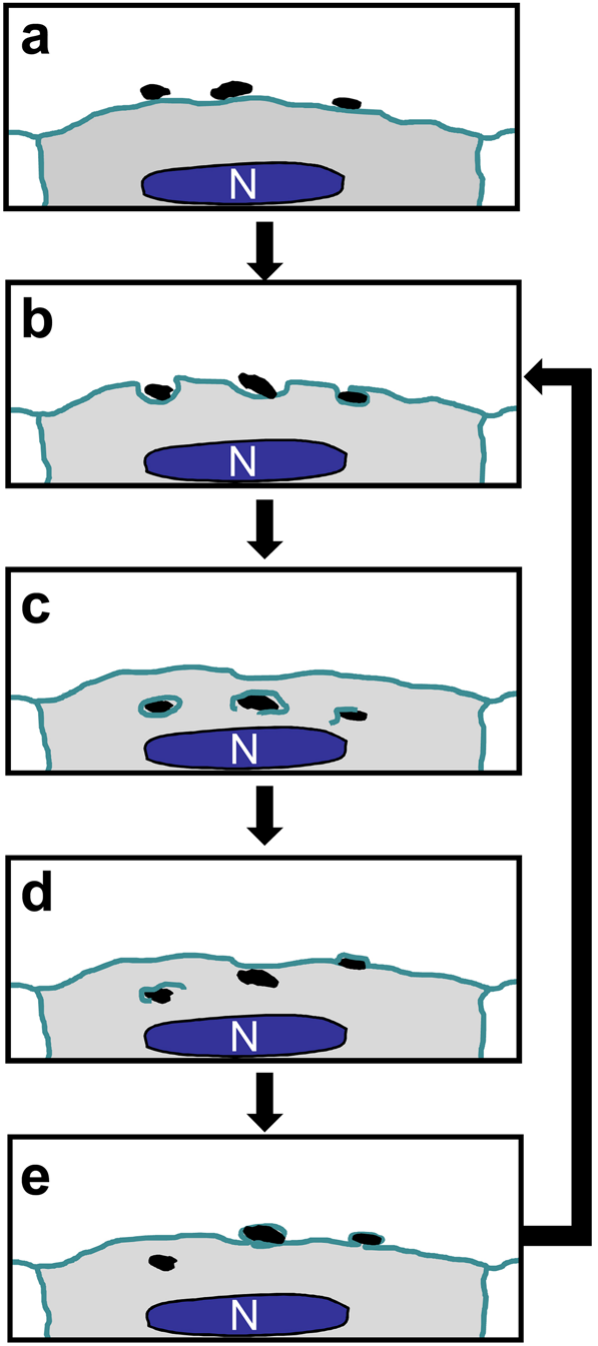
Scheme of endocytosis-exocytosis cycle. **a** MNPs interaction with cell membrane; **b** cell membrane invagination; **c** internalization on MNPs agglomerates in membrane vesicles; **d** lost of membrane and beginning of exocytosis; **e** exit of MNPs agglomerates to the cell surface. After last phase MNPs agglomerates can be re-absorbed by this or a neighboring cell and the cycle repeats (*arrow*)

Long-term live imaging of cells with MNPs allowed us to discover that a part of internalized MNPs with time reappears at the cell surface where they can be re-captured by the same or neighboring cell (Additional file 2: Movie 2). This MNPs “recycling” becomes more apparent when the external magnetic field was applied. In this case, MNPs agglomerates appearing at the cell surface are readily dragged towards the magnet (Fig. 4), similar to the whole MNP-loaded cells that lost contact with the substrate. Ultimately, after one-week incubation in magnetic field, the majority of cells had lost cytoplasmic MNPs (data not shown). The scheme of MNPs turn-over through endocytosis-exocytosis cycle is presented on Fig. 8.

## Conclusions

We demonstrated superparamagnetic properties and magnetocontrollability of Fe_7_C_3_@C MNPs in living cells. In combination with low cytotoxicity and high potential for chemical modification of carbon shell shown in our previous studies, these properties make the MNPs a promising candidate as a platform for targeted drug delivery. Enhanced magnetic properties of Fe_7_C_3_@C MNPs make it possible to control and concentrate MNPs efficiently at least at the centimeter scale, thus opening the opportunities to manipulate MNPs not only at cellular but also organismal level. Additional advantages of Fe_7_C_3_@C MNPs seem to be their early endosome escape into the cytoplasm that does not require additional efforts to facilitate drug release into cytoplasm and assures protection of carried substances from lysosomal degradation. The MNPs “recycling” would potentially allow magnetic field-assisted tissue clearance after drug release that would decrease side effects of therapeutic application of MNPs.

## Additional files

10.1186/s12951-016-0219-4 Live-cell imaging of HT1080 cells collecting MNPs agglomerates from glass surface. No magnetic field applied.
10.1186/s12951-016-0219-4 Live-cell imaging of HT1080 cells collecting MNPs agglomerates from the surface of neighboring cell. No magnetic field applied.
10.1186/s12951-016-0219-4 In moving HT1080 cell MNPs agglomerates keep their position in the magnet-proximal side of the cell independently of the direction of cell motility. Magnet is positioned below.
10.1186/s12951-016-0219-4 In moving HT1080 cell MNPs agglomerates keep their orientation along the magnetic field lines independently of the direction of cell motility. Magnet is positioned below.
10.1186/s12951-016-0219-4 The magnetic field favors the directionality of movement for cells heavily loaded with MNPs. Magnet is positioned below.
10.1186/s12951-016-0219-4 HT1080 cells dragged by the magnetic field force towards the magnet as a consequence of disassembly of microtubule and actin cytoskeletons. Magnet edge visible in the upper part of the frame.
